# Phage Therapy in Managing Multidrug-Resistant (MDR) Infections in Cancer Therapy: Innovations, Complications, and Future Directions

**DOI:** 10.3390/pharmaceutics17070820

**Published:** 2025-06-24

**Authors:** Alice N. Mafe, Dietrich Büsselberg

**Affiliations:** 1 Department of Biological Sciences, Faculty of Sciences, Taraba State University, Main Campus, Jalingo 660101, Taraba State, Nigeria; mafealice1991@gmail.com; 2 Weill Cornell Medicine-Qatar, Education City, Qatar Foundation, Doha Metropolitan Area, Ar-Rayyan P.O. Box 22104, Qatar

**Keywords:** phage therapy, multidrug-resistant (MDR) infections, cancer-associated infections, bacteriophage-based treatment, oncology infectious disease management

## Abstract

Multidrug-resistant (MDR) bacterial infections present a major challenge in cancer therapy, particularly for immunocompromised patients undergoing chemotherapy, radiation, or surgery. These infections often arise from prolonged antibiotic use, hospital-acquired pathogens, and weakened immune defenses, leading to increased morbidity and mortality. As conventional antibiotics become less effective against MDR strains, there is an urgent need for alternative treatment options. This review highlights phage therapy as a promising approach to managing MDR bacterial infections in cancer patients. Once widely used, phage therapy has recently regained attention as a targeted antimicrobial strategy that can specifically eliminate harmful bacteria while preserving the beneficial microbiota. Phages work by directly lysing bacteria, disrupting biofilms, and synergizing with antibiotics to restore bacterial susceptibility. These mechanisms make phage therapy especially appealing for treating infections that complicate cancer treatments. However, the clinical application of phage therapy faces challenges such as variability in phage–host interactions, regulatory hurdles, and immune responses in patients. This review identifies gaps in current research regarding the use of phage therapy for MDR infections in cancer patients. By examining recent innovations, therapeutic mechanisms, and associated limitations, we provide valuable insights into the potential of phage therapy for improving infection management in oncology. Future research should focus on refining phage delivery methods, assessing long-term safety, and exploring combination therapies to maximize clinical efficacy. Overcoming these challenges could position phage therapy as a valuable complement to existing antimicrobial strategies in cancer care.

## 1. Introduction

Multidrug-resistant (MDR) bacterial infections are increasingly problematic in cancer therapy, significantly complicating treatment outcomes and heightening mortality risks, especially in immunocompromised patients [1]. These infections arise from factors such as overuse of antibiotics, hospital-acquired pathogens, and immune suppression caused by chemotherapy, creating a serious barrier to effective treatment [2]. MDR infections in cancer patients are particularly concerning, as pathogens like *Pseudomonas aeruginosa*, *Klebsiella pneumoniae*, and *Staphylococcus aureus* develop resistance to multiple antibiotic classes, resulting in higher risks of sepsis, organ failure, and prolonged hospitalizations [3].

With conventional antibiotics often failing to address these resistant pathogens, there is an urgent need for alternative treatment strategies [4]. Phage therapy has emerged as a promising solution, offering high specificity in targeting MDR bacteria while minimizing damage to the beneficial microbiota [5]. Unlike antibiotics, bacteriophages are capable of attacking bacteria within biofilms, a significant issue in cancer patients with medical devices, surgical wounds, or pneumonia [6]. Furthermore, phage–antibiotic synergy has shown potential, where phages enhance the effectiveness of antibiotics, potentially overcoming bacterial resistance [7]. This review will examine the potential of phage therapy in managing multidrug-resistant (MDR) infections in cancer patients, focusing on its mechanisms, innovations, challenges, and prospects. By leveraging phages’ unique capabilities, this approach could transform the management of MDR infections in oncology, reducing reliance on antibiotics and helping to combat the growing threat of antimicrobial resistance.

## 2. Mechanisms of Phage Therapy in Overcoming MDR

### 2.1. Phage Therapy for MDR Pathogens

Phage therapy presents a targeted solution for multidrug-resistant (MDR) infections by specifically infecting and destroying bacterial strains without affecting beneficial bacteria [8]. Unlike broad-spectrum antibiotics, which target both harmful and beneficial microbes, phages act selectively on their bacterial targets, preserving the host microbiome [9]. This specificity is achieved through receptor-binding proteins (RBPs) on the phage surface that recognize distinct bacterial receptors, such as lipopolysaccharides (LPSs), teichoic acids, or outer membrane proteins [10]. Once bound to the bacterium, the phage injects its genetic material, triggering the lytic cycle, during which the bacterial machinery is hijacked to produce new phages, ultimately leading to bacterial cell lysis and the release of more phages [8]. These released phages then infect neighboring bacteria, amplifying the therapeutic effect [11].

**Figure 1:**
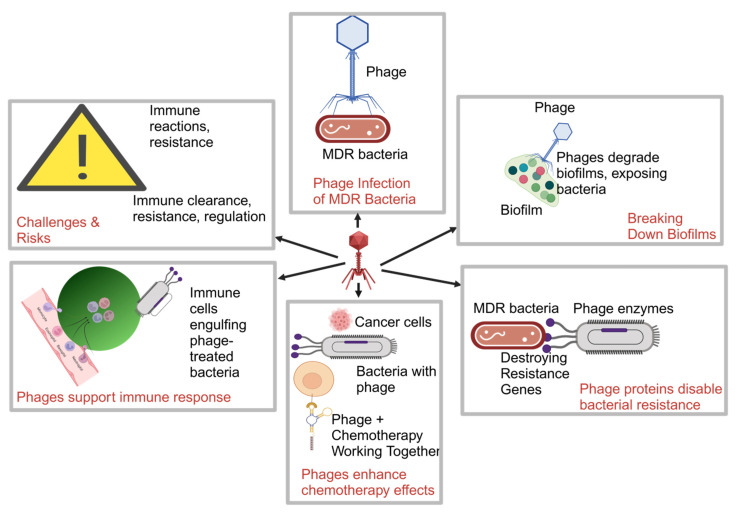
Mechanisms of phage therapy in overcoming MDR (created with BioRender). Key: Color coding (blue for phages, red for MDR bacteria, green for immune response, and yellow for chemotherapy.

In cancer patients, this precision in targeting MDR bacteria is especially valuable as it helps maintain the microbial balance critical for immune health and treatment success [12]. Phages are particularly effective against biofilm-associated infections, which are common in medical devices, catheters, and surgical wounds in oncology [13]. Phage therapy’s specificity, minimal off-target effects, and ability to self-amplify position it as a promising alternative to traditional antibiotics for treating recurrent MDR infections in cancer patients [14]. Phages can enhance the efficacy of chemotherapy by aiding bacterial clearance and stimulating immune responses [15]. Despite their potential, challenges like immune clearance and regulatory hurdles remain, necessitating further exploration to overcome these barriers. Figure 1 illustrates the mechanisms of phage therapy, highlighting key actions such as phage infection, biofilm disruption, chemotherapy synergy, immune support, and addressing risks like phage resistance and immune responses. The comparison of phage therapy and conventional antibiotics in managing MDR infections in cancer patients highlights core differences in target specificity, resistance development, biofilm penetration, microbiome impact, and effectiveness in immunocompromised patients (Table 1).

### 2.2. Key Mechanisms in Managing MDR Infections in Cancer Patients

#### 2.2.1. Targeted Lysis of MDR Bacteria in Immunocompromised Cancer Patients

Phage therapy is an effective strategy for combating multidrug-resistant (MDR) bacterial infections by targeting and lysing bacterial cells [30]. Unlike broad-spectrum antibiotics, which struggle against MDR strains due to resistance mechanisms like efflux pumps, enzymatic degradation, and target modifications, phages target bacteria specifically through their lytic cycle [31]. Upon binding to bacterial surface receptors, phages inject their genetic material into the cell, hijacking its machinery for replication [32]. This replication leads to the formation of new virions, triggering the production of endolysins, enzymes that degrade the bacterial cell wall, causing cell lysis and bacterial death [33]. This targeted approach minimizes disruption to the microbiome, which is particularly crucial for immunocompromised cancer patients, where maintaining a balanced microbiome is essential for overall health and immune function [34]. Since phages can co-evolve with bacteria, MDR pathogens find it harder to develop lasting resistance, unlike with antibiotics, where bacteria commonly evolve genetic mutations to evade treatment [35].

A key distinguishing aspect of phage therapy lies in the co-evolutionary interplay between bacteriophages and their bacterial hosts [36]. Unlike conventional antibiotics, which are static in nature, phages possess the unique ability to evolve in response to emerging bacterial resistance [37]. For instance, studies involving *Pseudomonas aeruginosa* have shown that when bacterial strains developed resistance to a specific lytic phage, the phage population was able to regain infectivity within 48 h through mutations in tail fiber proteins [38,39]. Likewise, in murine models infected with Escherichia coli, phages demonstrated adaptive mutations that restored their bactericidal activity shortly after resistance emerged [18,40]. In other cases, phages have acquired beneficial genetic elements through horizontal gene transfer, enabling them to circumvent bacterial defense mechanisms such as CRISPR-Cas systems [41,42]. These adaptive capabilities are evident in both in vitro assays and in vivo therapeutic models, indicating the evolutionary flexibility of phages, positioning them as a dynamic and sustainable strategy for combating multidrug-resistant (MDR) infections.

#### 2.2.2. Phage Disruption of Biofilms in MDR-Associated Cancer Infections

Biofilm formation significantly complicates MDR infections in cancer patients, primarily when associated with central venous catheters, urinary catheters, prosthetic implants, or ventilator-associated pneumonia [43]. Biofilms act as protective barriers, hindering antibiotic penetration and promoting the transfer of resistance genes [44]. Phages can disrupt these biofilms by producing enzymes such as depolymerases and hydrolytic enzymes, which break down the extracellular polymeric substance (EPS) matrix, effectively weakening the biofilm structure and enhancing bacterial vulnerability to immune responses and antibiotics [45]. These enzymes cleave the complex polysaccharides in the biofilm, facilitating the deeper penetration of antibiotics [46]. Phages can also infiltrate the biofilm to directly infect bacteria, spreading within the biofilm and gradually weakening the infection site [47]. This dual action of biofilm degradation and direct bacterial lysis offers a significant therapeutic advantage in treating MDR infections, particularly in cancer patients prone to device-related and surgical-site infections [48].

#### 2.2.3. Synergy with Antibiotics to Overcome MDR Infections

MDR infections often pose a significant challenge due to the ineffectiveness of conventional antibiotics in eliminating resistant bacterial populations [49]. Phage therapy offers a solution by inducing genetic changes in bacteria that restore their susceptibility to antibiotics [50]. This is known as phage–antibiotic synergy (PAS), where bacteria under selective pressure from phage infection alter their surface receptors to evade phage attack [51]. In doing so, they may lose critical resistance mechanisms such as efflux pumps or antibiotic-modifying enzymes, making them sensitive again to previously ineffective antibiotics [52]. For instance, studies have shown that phages can enhance the effectiveness of antibiotics like ciprofloxacin or rifampin against resistant *Pseudomonas aeruginosa* and *Klebsiella pneumoniae* [53]. Phages also facilitate antibiotic penetration by breaking down biofilms, enabling antimicrobial agents to penetrate deeper into bacterial colonies [54]. Quantitative data show that phage–antibiotic combinations significantly reduce bacterial load and infection severity in MDR infections, even in complex cases involving cancer patients [55]. For example, a study demonstrated that combining phages with vancomycin resulted in a 70% reduction in bacterial burden in *Staphylococcus aureus* infections [56]. This combination therapy boosts treatment efficacy and reduces the need for high antibiotic dosages, minimizing the risk of toxicity [57].

#### 2.2.4. Drawbacks of Phage Therapy Mechanisms in MDR Infections in Cancer Patients

Despite its potential, phage therapy for MDR infections in cancer patients faces several challenges. One key concern is the potential development of bacterial resistance to phages. Just as bacteria become resistant to antibiotics, they can evolve resistance to phages through mechanisms such as receptor mutations, CRISPR-Cas immunity, and the production of anti-phage defense proteins [58]. These resistance mechanisms may limit the long-term effectiveness of phage therapy, leading to the need for phage cocktails or genetically engineered phages to overcome bacterial adaptations [59]. Another challenge is phage clearance by the host immune system. Cancer patients undergoing chemotherapy or immunosuppressive treatments often exhibit altered immune responses, making phage therapy unpredictable [60]. In immunocompromised patients, the reduced immune response may lower the likelihood of rapid phage neutralization but could also lead to unchecked bacterial growth if phages fail to act effectively [61]. In contrast, patients with a partially intact immune system may quickly eliminate phages through neutralizing antibodies, complement activation, or phagocytosis, limiting their therapeutic potential [62].

**Figure 2:**
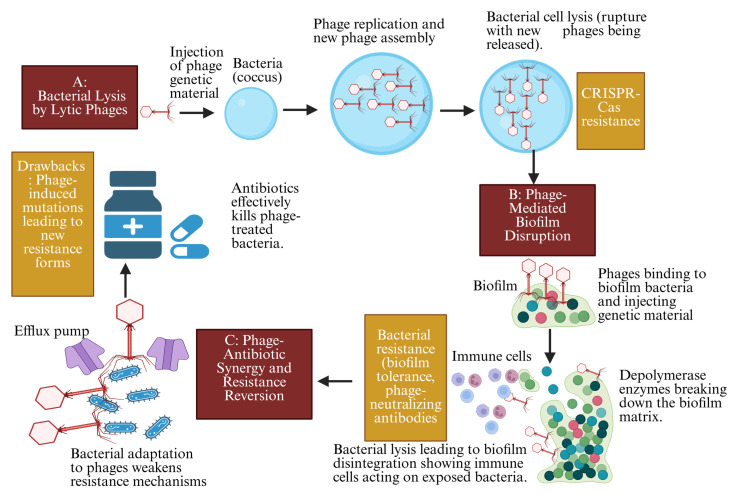
Mechanisms of phage therapy in combating bacterial infections and resistance: mechanism of bacterial lysis by lytic phages, phage-mediated biofilm disruption, and phage–antibiotic synergy and resistance reversion (created with BioRender).

Phage stability also presents an issue in treating MDR infections. Factors such as gastric acidity, enzymatic degradation, and inflammatory responses can diminish phage viability [63]. Moreover, cancer-related metabolic alterations and the tumor microenvironment may affect the ability of phages to target bacterial infections effectively [64]. To address these challenges, researchers are exploring optimized delivery methods, such as encapsulated phages or targeted nanocarriers, which could enhance phage stability and improve therapeutic outcomes [65]. These mechanisms highlight the potential of phage therapy in treating MDR-associated infections, particularly in cancer patients where antibiotic resistance complicates treatment (Figure 2).

### 2.3. Case Studies: Successful Applications of Phage Therapy in MDR Cancer-Related Infections

#### 2.3.1. Phage Therapy in MDR Bacteremia in Cancer Patients

Bacteremia is a severe and life-threatening complication often encountered in cancer patients, especially those undergoing chemotherapy or stem cell transplants, due to their compromised immune systems [66]. In a particular case, a leukemia patient developed a bloodstream infection caused by multidrug-resistant (MDR) *Klebsiella pneumoniae*, which was resistant to last-line antibiotics, including carbapenems [67]. Faced with limited therapeutic options, a personalized phage cocktail was administered alongside antibiotics. Remarkably, the patient showed substantial clinical improvement, and complete bacterial clearance was observed within weeks [68]. This case provides strong evidence of the potential of phage therapy in managing MDR bacteremia, particularly in oncology patients with limited alternatives to antibiotic treatment.

#### 2.3.2. Phage Therapy for MDR *Pseudomonas aeruginosa* Pneumonia

MDR pneumonia is a significant concern among cancer patients, particularly those who are on ventilators, as they are especially vulnerable to respiratory infections [69]. One case involved a chemotherapy patient who developed an MDR *Pseudomonas aeruginosa* pneumonia that was resistant to both colistin and cephalosporins [70]. As a last resort, phage therapy was introduced. After several doses, the patient’s bacterial load decreased significantly, and their respiratory function improved, ultimately leading to a full recovery [71]. This case highlights the potential of phage therapy in combating antibiotic-resistant respiratory infections in immunocompromised cancer patients, providing a promising solution when traditional antibiotics fail [72].

#### 2.3.3. Treatment of MDR *Staphylococcus aureus* Surgical Site Infections

Surgical site infections (SSIs) are common in cancer patients, particularly those who undergo tumor resections or reconstructive surgeries [73]. The treatment of MDR *Staphylococcus aureus* infections, including methicillin-resistant strains (MRSA), is particularly challenging in these settings [74]. A recent clinical trial demonstrated the use of phage therapy to treat post-surgical MDR *Staphylococcus aureus* infections in cancer patients. The results of the study showed a marked reduction in bacterial burden, accelerated wound healing, and restoration of antibiotic susceptibility, which significantly improved the overall treatment outcomes [75]. These findings suggest that phage therapy can be an effective adjunct to conventional antibiotics, providing an additional line of defense against multidrug-resistant (MDR) super-susceptible infections (SSIs) in oncology patients.

#### 2.3.4. Breakthrough Applications in MDR Infections Related to Cancer Therapy

Several groundbreaking case studies have demonstrated the success of phage therapy in treating multidrug-resistant (MDR) infections in cancer patients, offering a glimpse into the potential of this innovative treatment. One notable case involved a leukemia patient suffering from an MDR *Aciinetobacter baumannii* bloodstream infection. The infection was resistant to all available antibiotics, and in light of the severity of the condition, compassionate-use phage therapy was administered. The result was the complete eradication of the infection, and the patient experienced significant recovery [76]. This case demonstrates the effectiveness of phage therapy in treating infections resistant to all known antibiotics. In another instance, a cancer patient with an MDR *Pseudomonas aeruginosa* lung infection following chemotherapy received a combination of phage therapy and antibiotics. This dual treatment approach led to complete bacterial clearance and restored the patient’s lung function. The success of this treatment highlights the value of phage therapy as an adjunct to antibiotics, particularly for managing resistant respiratory infections in immunocompromised patients [77]. Several clinical trials involving genetically engineered bacteriophages targeting *Staphylococcus aureus* and *Escherichia coli* in immunocompromised cancer patients have demonstrated promising therapeutic outcomes [78]. In a Phase I/II trial conducted on patients undergoing chemotherapy and suffering from persistent *S. aureus* infections, the administration of phage therapy led to an increased infection resolution rate, with no serious adverse events reported [79]. Similarly, another study involving patients with MDR *E*. *coli*-associated urinary tract infections showed a significant reduction in bacterial load within 24 h of intravenous phage administration, with improved clinical outcomes [80]. These trials highlight the safety, tolerability, and efficacy of genetically modified phages, particularly in high-risk patient groups such as those undergoing immunosuppressive treatments. The low incidence of phage resistance and minimal adverse effects denote phage therapy’s potential as a complementary or alternative strategy to antibiotics in managing multidrug-resistant infections among cancer patients.

#### 2.3.5. Summary of Case Studies

The following table summarizes key case studies that demonstrate the successful application of phage therapy in treating multidrug-resistant (MDR) infections in cancer patients. The clinical case summaries of phage therapy for MDR infections in cancer patients are summarized in Table 2.

These case studies provide a strong foundation for expanding phage therapy applications in cancer-related multidrug-resistant (MDR) infections, offering hope for improved patient outcomes in cases of antibiotic resistance.

## 3. Innovations in Phage Therapy

Phage therapy has seen remarkable advancements in recent years, particularly in the context of multidrug-resistant (MDR) infections in cancer patients [86]. Innovations in delivery systems, synthetic biology, and combination therapies enhance its efficacy, in terms of phage stability, immune clearance, and resistance development [87]. These advancements pave the way for more precise and effective treatment options, particularly for immunocompromised cancer patients who are highly susceptible to MDR infections [88].

### 3.1. Novel Delivery Systems

Delivering phages effectively to infection sites within the complex physiological environment of cancer patients poses a major therapeutic challenge [89]. To address this, researchers have developed innovative encapsulation techniques that enhance phage stability and therapeutic performance [90]. Systems such as liposomes and nanoparticles act as protective carriers, shielding phages from enzymatic degradation and immune system clearance while enabling controlled, site-specific release [91]. These delivery platforms significantly improve phage survival in circulation and enhance their ability to target intracellular bacterial reservoirs, a frequent feature of MDR infections in oncology settings [92]. These novel delivery systems enhance the overall efficacy of phage therapy in cancer patients by improving bioavailability and precision.

### 3.2. CRISPR-Cas9 as a Genetic Tool for Targeting MDR Resistance Genes

CRISPR-Cas9, developed initially as a genome-editing tool, has emerged as a powerful strategy in combating multidrug-resistant (MDR) infections [93]. Unlike conventional approaches, CRISPR-Cas9 can be engineered into bacteriophages to identify and disrupt resistance genes in pathogenic bacteria selectively [94]. This targeted approach eliminates the resistant strains and minimizes the risk of resistance gene propagation through horizontal gene transfer [95]. Preclinical studies have demonstrated the successful use of CRISPR-enhanced phages to target genes such as blaNDM-1 in *E. coli* and mecA in *Staphylococcus aureus*, resulting in the selective eradication of MDR pathogens without affecting beneficial microbiota [96]. Although clinical applications are still in the early stages, initial findings indicate high precision and therapeutic potential. As research progresses, CRISPR-based phage therapy holds great promise as a personalized and targeted solution for managing MDR infections, particularly in immunocompromised cancer patients.

### 3.3. Synthetic Biology Advances

Synthetic biology is transforming phage therapy by enabling the design of genetically modified bacteriophages with enhanced therapeutic functions [97]. Engineered phages can be tailored to exhibit superior lytic activity, leading to faster and more effective elimination of MDR bacteria, a critical need in cancer patients with compromised immune systems [98]. By modifying phage components to reduce immunogenicity, researchers are also improving the safety profile of these therapies for oncology applications [99]. A key breakthrough involves the engineering of phages that produce enzymes, such as depolymerases, to degrade bacterial biofilms, which are often resistant to standard treatments and prevalent in cancer-related infections [20]. For instance, Dedrick et al. (2019) reported a clinical case in which a patient with a life-threatening Mycobacterium abscessus infection was successfully treated using a synthetic phage cocktail engineered for enhanced host specificity and lytic capacity [100]. Moreover, synthetic biology has facilitated the development of phage cocktails, which combine multiple engineered phages, thereby allowing for broader coverage against various bacterial strains [101]. This approach reduces the risk of bacterial resistance to single phages. It is currently being tested in clinical settings, including trials targeting MDR *Staphylococcus aureus* and *Pseudomonas aeruginosa* infections in immunocompromised cancer patients [102]. The integration of synthetic biology in phage therapy provides a powerful and adaptable platform for addressing the growing challenge of multidrug-resistant (MDR) infections in oncology, paving the way for more effective and personalized treatment strategies.

### 3.4. Combination Therapies

Combining phage therapy with other treatment strategies has emerged as a promising approach to address multidrug-resistant (MDR) infections, particularly in vulnerable cancer patients [103]. One of the most studied strategies is phage–antibiotic synergy (PAS), where phages enhance the effectiveness of antibiotics by weakening bacterial defenses [104]. This synergy can restore the activity of antibiotics that were previously ineffective, improving bacterial eradication and helping to reduce the emergence of further resistance [105]. However, recent advances go beyond simple antibiotic pairing. Innovative approaches now include combining phage therapy with immunotherapies used in cancer treatment [106]. Persistent bacterial infections can create an immunosuppressive tumor environment, limiting the success of immune-based cancer therapies. By clearing these infections, phages may help restore immune function and increase the effectiveness of treatments such as checkpoint inhibitors [107]. Researchers are also exploring the integration of phage therapy with targeted cancer therapies, chemotherapeutic agents, and novel drug delivery systems. These multimodal approaches aim to treat infection, improve the patient’s overall therapeutic response, and reduce treatment complications [108]. As research progresses, ongoing studies continue to evaluate the optimal combinations, timing, and delivery methods for using phage therapy alongside other medical interventions. These strategies promise to create more comprehensive and personalized treatment plans for cancer patients facing complex, drug-resistant infections [106].

Table 3 highlights crucial advancements, including encapsulation strategies, CRISPR-Cas9-engineered phages, and phage–antibiotic synergy, which have enhanced phage therapy’s stability, specificity, and effectiveness in managing MDR infections in cancer patients.

## 4. Complications and Limitations

Phage therapy holds great promise in responding to multidrug-resistant (MDR) infections in cancer patients; however, several complications and limitations must be addressed before its widespread clinical application [114]. These roadblocks include immune reactions, ethical concerns in personalized treatments, and logistical difficulties in hospital settings. Tackling these issues is essential to optimizing phage therapy for routine medical use.

### 4.1. Immune Reactions and Phage Stability

One of the primary challenges in implementing phage therapy, especially in cancer patients, is the body’s immune response to administered phages [115]. In immunocompromised individuals, such as those undergoing chemotherapy or radiation, the immune system is often altered, either too suppressed to assist therapeutic outcomes or capable of mounting a response that prematurely eliminates the phages before they reach their bacterial targets [116]. This immune recognition of phages as foreign particles can significantly reduce their efficacy, as antibodies or other immune components may neutralize them in circulation [117]. For cancer patients with varied immune statuses depending on disease progression and treatment protocols, the response to phage therapy is unpredictable and must be carefully managed [118]. To address this, advanced encapsulation strategies have been developed. Techniques involving liposomes or nanoparticles help protect phages from immune surveillance and enzymatic degradation, increasing their circulation time and improving delivery to the infection site [119]. Such innovations are particularly critical for targeting infections within immune-compromised and complex biological environments. In addition to immune clearance, the stability of phages inside the human body presents further challenges. Factors like fluctuations in pH, digestive enzymes, and interactions with host microbiota can reduce the viability of phages before they exert their therapeutic effect [120]. These challenges are even more pronounced in cancer patients, where delivering phages to hard-to-reach sites like tumor-associated infections is complicated by factors such as poor vascularization, immunosuppressive tumor microenvironments, and tissue heterogeneity [121]. Consequently, ensuring the stability and successful delivery of therapeutic phages in immunocompromised cancer patients remains a central focus of ongoing research to optimize treatment protocols and improve patient outcomes.

### 4.2. Ethical Considerations in Personalized Phage Therapy

The advancement of personalized phage therapy introduces critical ethical challenges that extend beyond its scientific potential [122]. Unlike traditional antibiotics, phage therapy often involves developing customized formulations tailored to a patient’s specific bacterial infection. While this precision approach enhances treatment effectiveness, it raises questions of equity, access, and regulatory oversight [123]. One pressing concern is the potential disparity in access to personalized phage treatments. The complex process of isolating, characterizing, and producing patient-specific phages can be time-consuming and expensive [124]. As a result, these therapies may be limited to well-resourced medical centers, making them less accessible to patients in low- and middle-income regions. This raises ethical questions about fairness and the potential widening of healthcare inequalities in the management of antimicrobial resistance [125]. Informed consent is another critical consideration. Patients undergoing phage therapy, particularly under experimental or compassionate use protocols, must be thoroughly informed about the nature of the treatment, including possible risks, benefits, and unknowns [126]. Full disclosure is critical when genetically modified (GM) phages are used, as these organisms are designed with enhanced properties that may raise concerns about safety and ecology [127]. The use of GM phages also presents ethical and regulatory challenges. Engineered to boost lytic activity or deliver gene-editing tools such as CRISPR-Cas systems, these phages offer promising therapeutic advantages [128]. However, the long-term impact on human health and microbial ecosystems remains unclear. Concerns about unintended consequences and environmental persistence have prompted cautious regulatory approaches and ongoing debate [129]. Ethical issues arise in the manufacturing and quality control of therapeutic phages. Ensuring safety, reproducibility, and compliance with Good Manufacturing Practice (GMP) standards is essential, especially when therapies are tailored on a case-by-case basis [130]. To integrate phage therapy responsibly into clinical practice, it is crucial to develop clear ethical guidelines and regulatory frameworks. These should promote equitable access, ensure rigorous safety evaluations, protect patient autonomy, and address public concerns regarding the use of genetically modified biological agents.

### 4.3. Logistical Complications in Hospital Settings

The practical implementation of phage therapy in hospital environments, particularly in oncology wards treating multidrug-resistant (MDR) infections, presents several logistical challenges. One significant issue is the storage and stability of phages [131]. Unlike antibiotics, which have well-defined storage conditions and shelf lives, phages require precise conditions to maintain their viability. Temperature fluctuations, contamination risks, and preparation time can all impact phage effectiveness [132]. Hospitals need robust diagnostic capabilities to rapidly identify bacterial strains and match patients with appropriate phage formulations. Current diagnostic processes can be time-consuming, delaying the initiation of treatment and reducing the therapeutic window of effectiveness. Streamlining bacterial profiling methods and developing standardized phage libraries will make phage therapy a viable option for multidrug-resistant (MDR) infections in oncology patients.

### 4.4. Case Examples: Problems in Clinical Trials

Several clinical trials and experimental applications of phage therapy have encountered significant obstacles, providing valuable lessons for future development. One notable example involved a trial investigating phage therapy for MDR *Pseudomonas aeruginosa* infections in cystic fibrosis patients [133]. Despite initial success in preclinical models, the trial was discontinued due to inconsistent efficacy in human subjects, likely stemming from rapid immune clearance and variability in bacterial susceptibility [134]. Another case involved a study on phage therapy for MDR *Escherichia coli* urinary tract infections. While some patients showed a reduction in bacterial load, others exhibited no clinical improvement, pointing out the challenge of patient-to-patient variability in phage responsiveness [135]. These findings underscore the need for improved patient stratification and optimized phage selection methods. Regulatory barriers have also impeded progress. A study exploring the use of engineered phages in oncology patients with bloodstream infections encountered significant delays due to stringent approval processes. The trial was eventually discontinued due to concerns over the safety of genetically modified phages, reflecting the bureaucratic hurdles that must be overcome before phage-based therapies can be widely adopted.

### 4.5. Overcoming Drawbacks: The Path Forward

To establish phage therapy as a mainstream treatment for MDR infections in cancer patients, several advancements are needed. Enhancing phage delivery methods, refining patient selection criteria, and expanding large-scale clinical trials will be preeminent. Focusing on the ethical concerns through regulatory frameworks and ensuring logistical feasibility in hospital settings will help bridge the gap between experimental research and real-world clinical applications [136]. Overcoming these stumbling blocks will be crucial in unlocking the full potential of phage therapy for managing MDR infections in immunocompromised cancer patients.

## 5. Critical Analysis of Current Literature

A thorough evaluation of the existing literature on phage therapy for multidrug-resistant (MDR) infections in cancer patients reveals promising advancements and significant research gaps. Although clinical evidence specific to oncology remains limited, with only a handful of case reports presented in this review, several broader studies in non-oncologic patients have demonstrated promising outcomes with phage therapy against multidrug-resistant (MDR) infections [137]. For example, Dedrick et al. (2019) successfully treated a disseminated *Mycobacterium* abscessus infection in a cystic fibrosis patient using engineered bacteriophages [100]. In another case, Tan et al. (2021) reported significant clinical recovery in a patient with a life-threatening *Acinetobacter baumannii* infection following a customized phage therapy regimen [138]. In addition, the PhagoBurn trial by Jault et al. (2019), although challenged by phage stability issues, demonstrated the feasibility of conducting controlled phage therapy trials in burn patients with *Pseudomonas aeruginosa* infections [139]. These studies serve as essential precedents and provide a foundational basis for exploring phage therapy applications in cancer patients, who often experience similar MDR infections. Nevertheless, variability in study design, limited large-scale clinical validation, and inconsistent outcomes indicate the need for more focused research within oncology. A critical evaluation of the current findings is, therefore, essential to assess their relevance for managing MDR infections in cancer therapy and to highlight key directions for future investigation.

### 5.1. Research Gaps and the Need for Large-Scale Clinical Trials

Although promising results have been observed in preclinical and early clinical studies, the absence of large-scale randomized controlled trials (RCTs) remains a significant obstacle to the broader implementation of phage therapy. Many existing studies have been conducted in vitro or in animal models, which do not always reflect the complexities of human clinical conditions [140]. This limits the translation of findings to actual patient care. A lack of consistency in research methodologies, including differences in phage strains, bacterial targets, and delivery methods, has led to varying outcomes across studies [141]. These inconsistencies complicate efforts to draw reliable conclusions and create standardized protocols for clinical use. As a result, regulatory approval and the clinical integration of phage therapy in oncology remain difficult without more rigorous and uniform data [137]. For phage therapy to be fully adopted in clinical practice, it is essential to conduct large-scale, multicenter randomized controlled trials (RCTs) that follow standardized protocols. These trials should focus on determining the optimal phage dosage, treatment schedules, and combinations with other therapies, such as antibiotics or immunotherapy [28]. Long-term safety and possible side effects should also be carefully evaluated. While phage therapy has demonstrated significant potential, substantial research gaps remain. Addressing these gaps through well-designed clinical trials is crucial to confirming its efficacy and paving the way for its widespread use in treating multidrug-resistant infections, particularly in cancer patients.

### 5.2. Conflicting Data on Phage Efficacy and Resistance Risks

While many studies support the effectiveness of phage therapy in targeting multidrug-resistant (MDR) bacterial strains, breaking biofilms, and enhancing antibiotic activity, there are inconsistencies regarding their long-term stability and immune interactions [142]. Some research demonstrates that phages can effectively target MDR bacteria and restore susceptibility to antibiotics, making them a promising treatment option in the fight against resistant infections [143]. However, other studies indicate that phages are often cleared from the bloodstream by the immune system, reducing their effectiveness in systemic infections [144]. Like antibiotics, bacterial resistance to phages has been reported in some instances, raising concerns about the sustainability of phage therapy. While solutions such as phage cocktails or genetic engineering aim to mitigate this issue, the potential for resistance remains a critical challenge [145]. Applying phage therapy within the tumor microenvironment is another area that requires further investigation. Some studies suggest that phages could enhance immune responses against infections in cancer patients. In contrast, others caution about unintended effects, such as interactions with immune cells or disruptions to the gut microbiota, which could impact cancer treatment outcomes [146]. To address these uncertainties, more in-depth research is needed to better understand how phages interact with the immunocompromised states of cancer patients and to explore strategies for overcoming resistance.

### 5.3. Future Directions in Oncology Applications

Despite the existing limitations, phage therapy presents a novel and adaptable strategy for contending with MDR infections in cancer patients, particularly those undergoing immunosuppressive treatments [14]. Integrating phage therapy with conventional cancer treatments, such as antibiotics and immunotherapy, may provide a multifaceted approach to infection management [24]. Using genetically engineered phages with enhanced lytic properties and reduced immunogenicity represents a promising avenue for overcoming current obstacles [9]. The potential of phage therapy to modulate microbial communities within the tumor microenvironment warrants further exploration, as emerging evidence suggests that the microbiome plays a role in cancer progression and treatment response [147]. To establish phage therapy as a viable intervention for MDR infections in oncology settings, future research should prioritize well-structured clinical trials, advanced delivery mechanisms, and a more comprehensive cognizance of phage–bacteria–host interactions [71]. Resolving these difficulties will be critical in positioning phage therapy as a transformative tool in MDR cancer therapy and improving patient outcomes in oncology hospitals [15].

## 6. Future Work and Potential Directions

**Figure 3:**
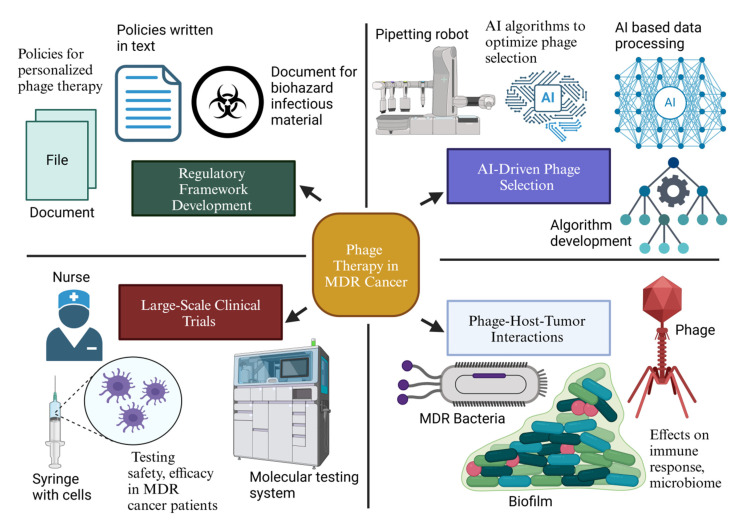
Proposed research roadmap for phage therapy in MDR cancer therapy (created with BioRender).

The future of phage therapy in multidrug-resistant (MDR) cancer-related infections hinges on cutting-edge innovations, targeted research, and strategic policy advancements. While current studies highlight phage therapy’s potential, overcoming its limitations requires interdisciplinary collaboration, technological advancements, and regulatory adaptations [148]. This section outlines promising future directions to enhance phage therapy’s efficacy, safety, and clinical applicability in oncology. Future research should prioritize AI-driven phage selection for personalized treatment, phage–host–tumor interactions, and comprehensive clinical trials to advance its clinical application. Regulatory reforms and interdisciplinary collaborations are essential to overcoming barriers and ensuring widespread adoption (Figure 3).

### 6.1. Innovative Approaches

Recent breakthroughs in phage therapy research are revolutionizing the approach to treating multidrug-resistant (MDR) infections, particularly in oncology settings. Integrating artificial intelligence (AI) into the phage selection process is one such innovation. AI-driven algorithms can predict bacterial resistance patterns, allowing for the precise selection of phages and identification of the most effective phage combinations for individual cancer patients [149]. This personalized approach can significantly improve therapeutic outcomes by tailoring treatments to patients’ unique microbiological and immunological profiles. AI also plays a crucial role in rapidly identifying and engineering phages with enhanced antibacterial properties, ensuring greater efficacy against resistant infections [150]. Another significant development in phage therapy is the use of multi-phage cocktails [151]. Unlike single-phage treatments, which the development of bacterial resistance may limit, phage cocktails target multiple bacterial strains simultaneously, broadening the spectrum of activity [152]. This strategy reduces the risk of resistance and enhances treatment effectiveness, particularly in immunocompromised cancer patients who are more vulnerable to MDR infections [153]. Exploring the interactions between phages and bacterial quorum sensing mechanisms, as well as biofilm structures, is opening new doors to improving phage-based therapies [154]. Understanding these interactions could provide critical insights into optimizing phage treatments for persistent infections, particularly in cases related to cancer therapy.

### 6.2. Future Research Directions

A notable research gap in phage therapy is understanding its potential role in modifying the tumor microenvironment [155]. Although phages are primarily studied for their antibacterial properties, emerging evidence suggests they could also influence immune responses within tumors [156]. Future research should focus on determining whether phage therapy can stimulate anti-tumor immunity, alleviate inflammation, or affect the gut microbiome in ways that complement cancer treatments [157]. Extensive long-term safety and efficacy studies in oncology patients are essential [158]. Robust clinical trials are required to establish optimal dosing regimens, immune responses, and the sustained effects of repeated phage treatments. Without large-scale, well-designed trials, securing regulatory approval for phage therapy remains challenging [159]. Currently, large-scale clinical trials investigating phage therapy in oncology are limited. Such trials should aim to standardize treatment protocols, identify potential adverse effects, and evaluate the effectiveness of phage therapy for various cancer-associated MDR infections [160]. Moreover, regulatory frameworks will need to adapt to the personalized nature of phage treatments [161]. Existing guidelines, which are primarily tailored for traditional antibiotics, may not be suitable for phage therapy’s unique characteristics [162]. Therefore, policymakers should consider more flexible clinical trial designs, compassionate use protocols, and regulatory adjustments that better accommodate personalized phage-based treatments.

### 6.3. Recommendations

Collaboration across multiple disciplines is crucial for transitioning phage therapy from research to clinical practice. Researchers, clinicians, microbiologists, and bioengineers must collaborate to address key challenges, including phage stability, immune system interactions, and large-scale production. The formation of global research consortia focused on phage therapy could help facilitate knowledge sharing and accelerate progress in this field. Regulatory reforms are vital for speeding up the approval process for phage therapies [79]. Given that phage treatments are highly personalized, existing regulatory frameworks must be updated to accommodate these therapies better. Policymakers should support flexible regulatory approaches that enable precision phage treatments, streamline clinical trials, and expand compassionate use programs for patients with life-threatening MDR infections. Implementing these strategies could enhance treatment outcomes for cancer patients by providing more effective and targeted therapies. By addressing current limitations and embracing emerging scientific advancements, phage therapy has the potential to transform infection management in cancer care, ushering in a new era of personalized antimicrobial treatment.

## 7. Conclusions

Phage therapy offers a promising alternative for treating multidrug-resistant (MDR) infections in cancer therapy, representing an innovative and evolving approach to combat bacterial resistance. This review has explored the various mechanisms by which phages target resistant bacteria, including direct bacterial lysis, disruption of biofilms, and their synergistic effects with antibiotics. Exciting advancements, including AI-assisted phage selection, nanoparticle-based delivery systems, and CRISPR-enhanced phages, have significantly expanded the therapeutic possibilities of phage therapy. However, several key challenges remain, including immune clearance, regulatory hurdles, and a limited understanding of phage–host–tumor interactions within the tumor microenvironment. A thorough examination of the current literature reveals significant progress and research gaps, underscoring the need for large-scale clinical trials, further investigation into phage-immune dynamics, and the development of standardized treatment protocols. While phage therapy shows great promise, overcoming its challenges will require significant advancements in synthetic biology, AI-guided phage engineering, and stronger interdisciplinary collaboration. The integration of phage therapy with immunotherapy, precision antibiotics, and tumor-targeted strategies could markedly improve treatment outcomes for cancer patients battling MDR infections. Addressing these challenges requires a coordinated effort among researchers, clinicians, bioengineers, and policymakers. Future research should prioritize the development of flexible regulatory frameworks to facilitate the clinical application of personalized phage therapy. Moreover, investing in advanced computational tools and high-throughput screening will be crucial in optimizing phage selection and therapeutic efficacy. Understanding the complex interactions between phages, the immune system, and the tumor microenvironment will be essential to unlocking the full potential of phage therapy. Integrating phage therapy into MDR cancer treatment could revolutionize infection management, reduce reliance on conventional antibiotics, and contribute to global efforts to combat antimicrobial resistance. By addressing existing research gaps, embracing technological advancements, and streamlining regulatory processes, phage therapy could become a transformative tool in modern cancer care.

## Figures and Tables

**Table 1:** Comparative features of phage therapy vs. conventional antibiotics.

Feature	Phage Therapy	Conventional Antibiotics
Target Specificity	High; targets specific bacterial strains, e.g., K. *pneumoniae*-specific phages used in bacteremia cases [16]	Broad or narrow spectrum; may kill both pathogenic and beneficial bacteria [17]
Resistance Development	Low; phages co-evolve with bacterial mutations; resistance is often transient and manageable [18]	High bacterial resistance (e.g., MRSA, ESBL-*E. coli*) is escalating globally [19]
Biofilm Penetration	Highly effective; phage-derived depolymerases degrade biofilms, e.g., shown in *P. aeruginosa* lung infections [20]	Limited; antibiotics often fail to penetrate biofilm matrices, leading to relapse [21]
Impact on Microbiome	Minimal; preserves commensals; shown to reduce dysbiosis in murine studies [22]	Significant; alters gut flora; may cause *C. difficile* overgrowth and secondary infections [23]
Effectiveness in Immunocompromised Patients	Promising; successful in leukemia and chemotherapy patients; efficacy may improve with adjunctive immune support [24]	Often reduced; due to high resistance and microbiome disruption, e.g., failure of colistin in neutropenic patients [25]
Immune Response and Side Effects	Mild; generally well tolerated; occasional immune neutralization may limit dosing [26]	Variable; allergic reactions, nephrotoxicity, and hepatotoxicity are common with drugs like aminoglycosides [27]
Regulatory Approval and Clinical Use	Limited; in Phase I/II trials; FDA-approved for compassionate use; lacks standardized protocols [28]	Established; broad clinical use, regulatory approval, and dosing guidelines worldwide [29]

**Table 2:** Clinical case summaries of phage therapy for MDR infections in cancer patients.

Patient Summary	Infection Type	Pathogen (Resistance Profile)	Phage Type	Mode of Delivery	Therapy Duration	Adjunct Therapy	Clinical Outcome and Follow-Up
A leukemia patient undergoing immunosuppressive therapy	Bloodstream infection	*Klebsiella pneumoniae* (MDR)	Personalized phage cocktail	Intravenous infusion	7 days	Carbapenem antibiotic	Complete bacterial clearance with significant clinical recovery; sustained response at 30-day follow-up [81]
A chemotherapy patient with neutropenia	Pneumonia	*Pseudomonas aeruginosa* (MDR)	Natural lytic phage	Inhalation (nebulizer)	10 days	None	Marked reduction in bacterial load; full respiratory recovery noted within 2 weeks [82]
Post-operative cancer patient	Surgical site infection	*Staphylococcus aureus* (MRSA)	Phage cocktail	Topical + Intravenous	14 days	Aminoglycoside	Faster wound healing observed; bacterial susceptibility to antibiotics restored post-therapy [83]
Advanced lung cancer patient post-chemotherapy	Lung infection	*Pseudomonas aeruginosa* (MDR)	Natural + Engineered phage combo	Inhalation + IV	10–14 days	Colistin and immune support	Complete bacterial eradication and restored lung function; follow-up confirmed sustained recovery [77]
Solid tumor patient with sepsis	Bloodstream infection	*Acinetobacter baumannii* (MDR/XDR)	Compassionate-use phage therapy	Intravenous	7–10 days	Tigecycline	Pan-resistant infection eradicated; notable systemic improvement and no adverse effects [84]
Colorectal cancer patient	Urinary tract infection	*Escherichia coli* (ESBL+)	Engineered phage (clinical trial)	Intravesical instillation	5 days	None	Infection resolved with minimal adverse effects; no recurrence observed during 30-day monitoring [85]

**Table 3:** Innovations in phage therapy and their advantages.

Innovation	Description	Impact on Treating MDR Infections
Liposome and Nanoparticle Delivery	Encapsulation of bacteriophages in liposomes or polymeric nanoparticles for targeted delivery	Enhances phage stability and bioavailability, enabling effective delivery to MDR infection sites [109]
CRISPR-Cas9-Enhanced Phages	Engineered phages integrated with CRISPR-Cas9 systems to cleave bacterial DNA at specific loci	Provides precise genome targeting, significantly reduces resistance development in MDR pathogens [110]
Synthetic Biology Phages	Phages modified using synthetic biology to improve lysis capability and reduce immunogenicity	Boosts therapeutic efficiency and host compatibility against MDR bacteria [111]
Phage–Antibiotic Synergism	Strategic combination of phages with conventional antibiotics for enhanced antibacterial effects	Restores or enhances antibiotic efficacy, overcoming resistance in MDR strains [112]
Integration with Immunotherapy	Utilization of phages to stimulate or modulate host immune responses during infection control	Offers potential synergy with immunotherapies for better clearance of MDR infections and biofilms [113]

## Data Availability

No new data were created or analyzed in this study. Data sharing is not applicable to this article.

## Abbreviations

The following abbreviations are used in this manuscript:

MDR	Multidrug-Resistant
RBPs	receptor-binding proteins
LPS	Lipopolysaccharides
EPS	extracellular polymeric substance
CRISPR-Cas	Clustered Regularly Interspaced Short Palindromic Repeats—CRISPR-associated proteins
SSIs	Surgical site infections
MRSA	methicillin-resistant *Staphylococcus aureus*
PAS	Phage–antibiotic synergy
GM	genetically modified
RCTs	randomized controlled trials
